# CRISPR/Transposon gene integration (CRITGI) can manage gene expression in a retrotransposon-dependent manner

**DOI:** 10.1038/s41598-019-51891-6

**Published:** 2019-10-25

**Authors:** Miki Hanasaki, Hiroshi Masumoto

**Affiliations:** Biomedical Research Support Center (BRSC), Nagasaki University School of Medicine, 1-12-4 Sakamoto, Nagasaki, Nagasaki 852-8523 Japan

**Keywords:** Genetic engineering, Expression systems

## Abstract

The fine-tuning of gene expression contributes to both basic science and applications. Here, we develop a novel gene expression technology termed CRITGI (CRISPR/Transposon gene integration). CRITGI uses CRISPR/Cas9 to integrate multiple copies of the plasmid pTy1 into Ty1 loci, budding yeast retrotransposons. The pTy1 plasmid harbors a Ty1 consensus sequence for integration, a gene of interest with its own promoter and a selection marker gene. Interestingly, the expression of the pTy1 gene in Ty1 loci could be induced in synthetic complete amino acid depletion medium, which could activate the selection marker gene on pTy1. The induction or repression of the gene on pTy1 depended on Ty1 transcription. Activation of the selection marker gene on pTy1 triggered Ty1 transcription, which led to induction of the gene on pTy1. The gene on pTy1 was not transcribed with Ty1 mRNA; the transcription required its own promoter. Furthermore, the trimethylation of histone H3 on lysine 4, a landmark of transcriptionally active chromatin, accumulated at the 5′ end of the gene on pTy1 following selection marker gene activation. Thus, CRITGI is a unique gene regulation system to induce the genes on pTy1 in amino acid depletion medium and utilizes Ty1 transcription to create a chromatin environment favorable for the transcription of the genes on pTy1.

## Introduction

Clustered regularly interspaced palindromic repeats (CRISPR) have evolved as a primitive immune system in prokaryotes with the ability to precisely target and edit any genome^1–4^. The Cas9 endonuclease of the Class II CRISPR system initially binds to a single-stranded RNA (single guide (sg) RNA) containing a short stretch of RNA (~20 bases) that binds and recruits the Cas9/RNA complex to the corresponding sequence within a target genome locus that is also marked with a protospacer adjacent motif (PAM) 5′-NGG-3′ sequence^5,6^. The Cas9/sgRNA complex causes a double-stranded break (DSB) at the position upstream of the PAM. DSBs introduced at the genome position(s) by the Cas9/sgRNA complex have enabled gene replacement, gene deletions and single base editing in many eukaryotic organisms, including humans^7–11^. *Saccharomyces cerevisiae*, or budding yeast, is one of the most studied and genetically and biologically tractable organisms. The tractability of yeast in both basic science and industry stems from the ability to rapidly edit and manipulate its genome. Together with the evolved CRISPR system suitable for budding yeast cells, various efforts have provided a new suite of molecular tools using the CRISPR system that have been applied to a diverse array of methodologies in budding yeast, including multiplexed editing, chromosome splitting, transcriptional modulation, synthetic genome engineering and gene drive technology^12–17^.

## Results and Discussion

**Figure 1:**
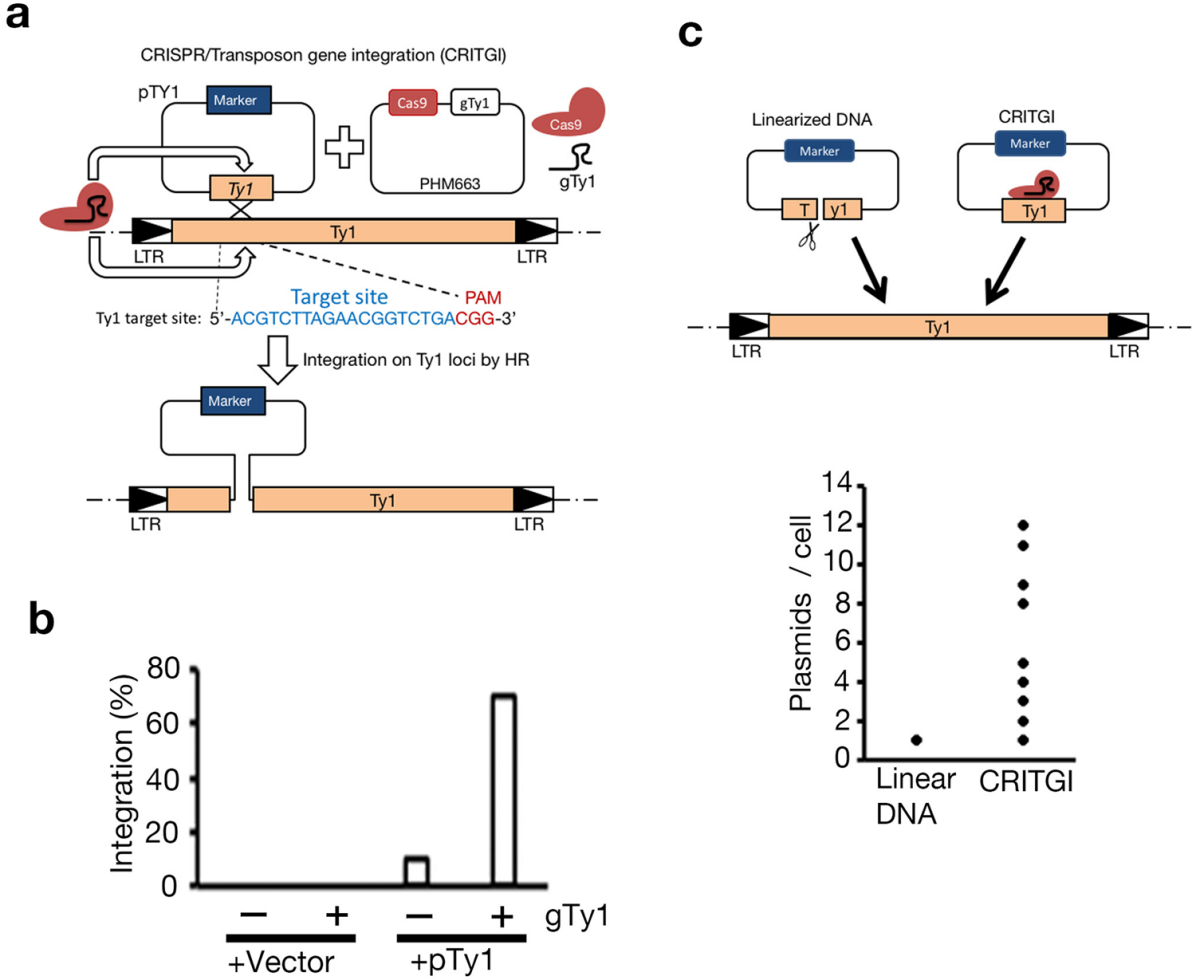
CRISPR/Transposon gene integration (CRITGI) can introduce multiple plasmids into Ty1 loci. (**a**) A diagram of CRITGI. Two types of plasmids (pTy1 and PHM663) were simultaneously transformed into yeast. Blue colored bases and red colored bases indicate the target sequence and 5′ PAM sequence of gTy1, respectively. Marker: marker gene for selection of transformants. (**b**) Percentage of correct integration of the pTy1 plasmid into Ty1 loci. YIplac128 was used as a vector. (**c**) Comparison of the integrated pTy1 plasmid number in Ty1 loci between the linear plasmid and CRITGI. The *Sal* I-digested linear pTy1 plasmid (linear DNA) and the mixture with the pTY1 and PHM663 plasmids (CRITGI) were transformed into yeast cells. The number of integrated pTY1 plasmids on Ty1 loci was calculated for the *ACT1* gene as one copy gene per genome by using real time (RT)-PCR, which included transformants derived from linear DNA (n = 8) and those from CRITGI (n = 20).

In this research, our first goal was to develop a novel gene expression tool using the CRISPR/Cas9 system suitable for budding yeast. To obtain a strain with stable expression of the protein of interest, we usually select a strategy to integrate a linearized expression vector into the target locus in the chromosome by homologous recombination (HR)^18^. Multiple integrations of an expression vector are expected to increase the expression of the protein of interest. However, we usually obtain a strain harboring one copy of a plasmid in a chromosome, even though many target loci exist in the chromosome. To establish a method for multiple integrations of an expression vector, we tested the CRISPR/Cas9 system to introduce multiple copies of a plasmid into multiple target loci in the yeast genome. We used Ty1, one of five yeast retrotransposons (Ty1 to Ty5), as which is located at multiple sites throughout the genome; 31 are complete forms, and truncated forms occupy 3% of the total genome volume^19,20^. The coding region of Ty1 is flanked by two long terminal repeats (LTRs) that function as the transcriptional promoter and terminator (reviewed in^20^). Shi and colleagues have already developed Di-CRISPR to integrate multiple copies of gene clusters into the LTR regions of Ty1 and Ty2 (delta) sites^12^. We used the sequence region from 1682 bp to 2518 bp (836 bp length) of *YPRWRTy1-3*, named the Ty1 HR sequence, as a vector integration site, which is highly conserved among the 31 Ty1 complete forms^21^. A mixture of the integration vector harboring the Ty1 HR sequence (pTy1) and PHM663 plasmid expressing Cas9 and sgRNA to recognize the Ty1 HR sequence (gTy1) was transformed into yeast cells. The Cas9/gTy1 complex can introduce DSBs within the Ty1 HR sequence in the pTy1 plasmid and then integrate the plasmid into the Ty1 locus by HR (Fig. 1a). We named this plasmid integration system CRISPR/Transposon gene integration (CRITGI). CRITGI required the Ty1 HR sequence in the pTy1 plasmid to obtain transformants; the transformation efficiency of the pTy1 plasmid significantly dropped without the Ty1 HR sequence (Fig. S1). gTY1 sgRNA was required for pTy1 plasmid integration into Ty1 loci with 75% accuracy, in contrast to 10% accuracy without gTy1 (Fig. 1b). Furthermore, direct sequencing revealed that the sequence at the pTy1 plasmid-integration site on the Ty1 locus in all transformants (n = 10) was identical to the reference sequence of the parent strain (BY4742) (Fig. S2), suggesting that the pTy1 plasmid was correctly integrated into the Ty1 locus by HR. Multiple DSBs by Cas9/gTY1 in Ty1 loci might trigger rearrangement among chromosomes. Pulse field gel electrophoresis (PFGE) showed that the size of any chromosome band had no obvious change in transformants compared to wild type (Fig. S3), suggesting that CRITGI could integrate the pTy1 plasmid without large-scale genomic rearrangement. Next, we used quantitative PCR (qPCR) to count the copy number of pTy1 plasmids in the transformants. Although transformation using linearized pTy1 plasmid resulted in a strain with a single copy of the pTy1 plasmid in the Ty1 locus (Fig. 1c; Linear DNA), CRITGI could introduce multiple copies of pTy1 plasmids into Ty1 loci (Fig. 1c: CRITGI). Thus, CRITGI enables multiple copy numbers of pTy1 plasmids to integrate into Ty1 loci, as in Di-CRISPR^12^. Furthermore, we examined whether CRITGI simultaneously enabled several different types of pTy1 plasmids (3 species) to integrate into Ty1 loci. We obtained transformants with several species of pTy1 plasmids, in which the frequency of plasmid combination was reduced to 50% (2 pair) and 10% (3 pair) among the total transformants examined (n = 10) (Fig. S4D). Thus, CRITGI is able to introduce not only multiple copies of pTy1 plasmids but also various species of pTy1 plasmids into Ty1 loci.

**Figure 2:**
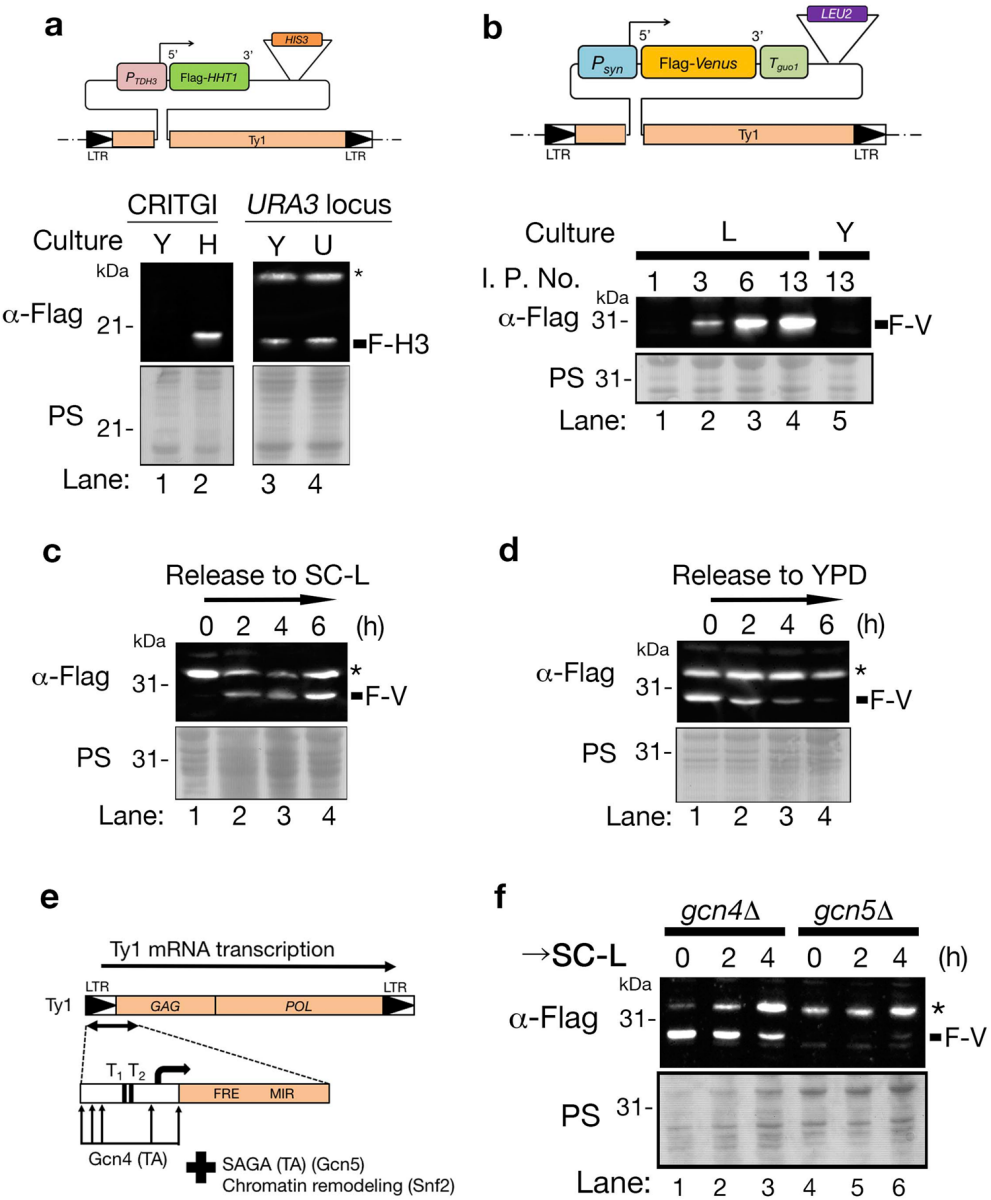
CRITGI regulates protein expression in a Ty1-dependent manner. (**a**) Flag-H3 expression in cells with the pTy1-H3 plasmid was induced in medium to activate the marker gene. A schematic representation of the pTy1-H3 plasmid integrated into the *Ty1* locus (top). The FLAG-*HHT1* gene encodes Flag-histone H3 (F-H3). *P*_*TDH3*_: *TDH3* promoter. *HIS3*: *HIS3* marker gene. Immunoblotting using an anti-Flag antibody detected Flag-H3 in cell extracts (bottom). Cells were cultured in either synthetic complete (SC) histidine dropout media (SC-His) or YPD media at 25 °C overnight. CRITGI: HMY1466 strain (wild-type cells with the pTy1-H3 plasmid (I. P. No. = 11)). *URA3* locus: HMY1502 strain harboring a single copy of Flag-histone H3 with the *TDH3* promoter in the *URA3* locus. I. P. No.: integrated plasmid number. Y: YPD culture. H: SC-His culture. U: SC-Ura culture. PS: Ponceau S staining. *Nonspecific protein band. Cell extracts were analyzed by immunoblotting using α-Flag antibody. (**b**) The expression level of Flag-Venus increased proportionally with the integrated plasmid number in Ty1 loci. A schematic representation of the pTy1-V plasmid integrated into the Ty1 locus (top). The pTy1-V plasmid harbors the Flag-Venus gene with the *Psyn* promoter, *Tguo1* terminator, and *LEU2* marker gene. Four types of HMY1494 strains (wild-type cells with the pTy1-V plasmid (I. P. No. = 1, 3, 6 and 13)) were cultured in YPD at 25 °C and then released into either SC-L or YPD. L: SC-Leu culture. F-V: Flag-Venus. (c and d) Immunoblot detection of Flag-Venus protein over time. The HMY1476 strain (wild-type cells with the pTy1-V plasmid (I. P. No. = 10)) was employed. (**c**) The HMY1476 strain was cultured in YPD at 25 °C and then released into SC-L. (**d**) The HMY1476 strain was cultured in SC-L at 25 °C and then released into YPD. (**e**) A schematic representation of Ty1 transcriptional regulation modified from Servant, G. *et al*.,^33^. *GAG* and *POL* are analogs to the retrovirus genes *GAG* and *POL*, respectively. T: TATA box sequence. TA: transcriptional activator. (**f**) Flag-Venus expression was influenced by trans-acting factors for Ty1 transcription. The HMY1491 strain (*gcn4*∆ with pTy1-V plasmid (I. P. No. = 24)) and HMY1492 strain ((*gcn4*∆ with pTy1-V plasmid (I. P. No. = 32)) were cultured in YPD at 25 °C and then released into SC-L (→SC-L).

**Figure 3:**
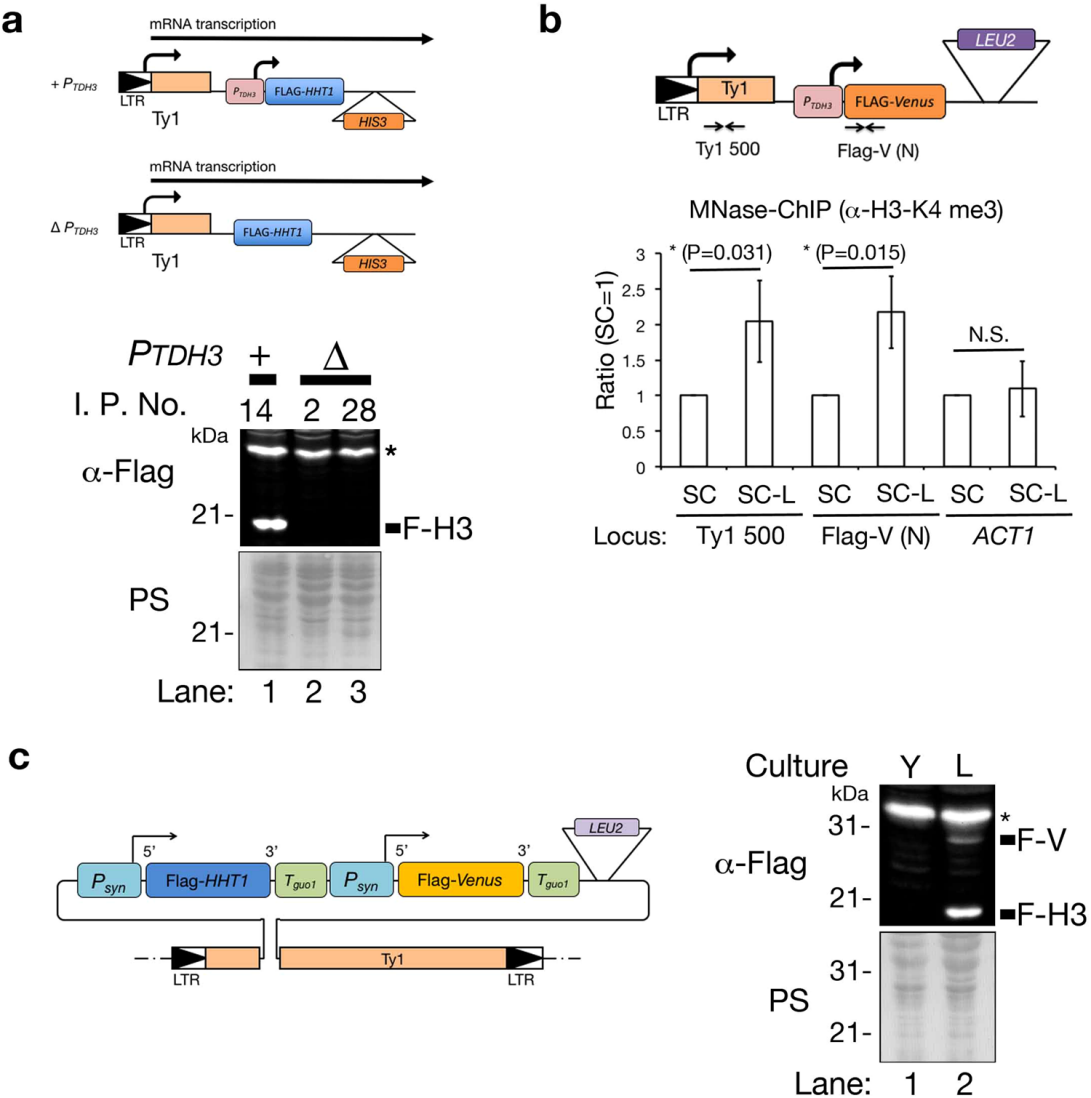
CRITGI promotes a chromatin structure favorable for gene expression. (**a**) A schematic representation of the FLAG-*HHT1* gene integrated into the Ty1 element in the presence and absence of *P*_*TDH3*_ (+*P*_*TDH3*_ and ∆*P*_*TDH3*_, respectively) (top). Immunoblotting using an anti-Flag antibody detects Flag-H3 in cell extracts (bottom). Two types of HMY1496 strains (wild-type cells with the pTy1-H3 ∆p plasmid (I. P. No. = 2 and 28, respectively)) and the HMY1466 strain (wild-type cells with pTy1-H3 (I. P. No. = 14)) were cultured in SC-His at 25 °C overnight. As in Fig. 2, the nitrocellulose membrane, in which proteins had been transferred to, was stained with 0.1% Ponceau S buffer and scanned to quantify the protein level in each lane. After destaining the membrane by TBS-T, the same membrane was used for the Western blot using the α-Flag antibody. (**b**) MNase-ChIP assay using anti-histone H3-K4me3 antibody. The HMY1476 strain (wild-type cells with the pTy1-V plasmid (I. P. No. = 4)) was cultured either in SC or in SC-Leu (SC-L) at 25 °C for 5 h. Ty1 500 and Flag-V (N) were used as target sites for RT-qPCR analysis. *P < 0.05. Unpaired t-test (one-tail). Error bars represent the standard deviation of three biological replicates. The *ACT1* gene locus was used as a control, and the H3-K4me3 level was not altered between SC and SC-L media. (**c**) CRITGI can synchronously express two types of genes set in tandem within Ty1 loci in SC-Leu, but not in YPD medium. A schematic representation of the pTy1-H3-V plasmid in the Ty1 element. The pTy1-H3-V plasmid harbors two gene sets: the FLAG-*HHT1* gene with the *Psyn* promoter and *Tguo1* terminator, FLAG-*Venus* gene with the *Psyn* promoter and *Tguo1* terminator in tandem with the *LEU2* marker gene (top). The HMY1500 strain (wild-type cells with the pTy1-H3-V plasmid (I. P. No. = 4)) was cultured either in SC-Leu (L) or YPD (Y) at 25 °C overnight. Immunoblots detected Flag-histone H3 (F-H3) and Flag-Venus (F-V) in cell extracts (bottom).

Several studies have claimed that the gene inserted in the Ty1 element may undergo transcriptional cosuppression, which is defined as high gene copy number-triggered homology-dependent gene silencing^22,23^. Therefore, we examined whether a gene in the pTy1 plasmid within Ty1 loci was able to express or not. The pTy1-H3 plasmid harbors the *HHT1* gene encoding the histone H3 protein with a FLAG epitope tag at the N-terminus driven by the strong constitutive *TDH3* promoter (Fig. 2a: top)^24^. An immunoblot using an α-FLAG antibody showed that either a faint or undetectable level of FLAG-H3 was expressed in cells with various copies of pTy1-H3 plasmid grown in yeast extract-peptone-dextrose (YPD) medium, a conventional rich medium (Fig. S5A,B). These results suggest that FLAG-H3 expression is downregulated within the Ty1 element. The pTy1-H3 plasmid harbors the *HIS3* gene as a marker gene, which is activated in cells grown in synthetic complete histidine dropout medium (SC-His). We hypothesized that activation of the *HIS3* gene might induce Flag-*HHT1* gene transcription from the pTy1-H3 plasmid. To test this hypothesis, we examined the expression of the FLAG-*HHT1* gene in cells with pTy1-H3 grown in SC-His medium. Surprisingly, FLAG-H3 was detected in cells with pTy1-H3 grown in SC-His but not in YPD (Fig. 2a (CRITGI); lanes 1 and 2), although FLAG-H3 expression was detected in both SC-Ura (uracil dropout) and YPD using cells with the FLAG-*HHT1* gene and the *TDH3* promoter integrated in the *URA3* locus (Fig. 2a (*URA3*); lanes 3 and 4). Thus, marker gene activation triggers the expression of the gene in cells with the pTy1 plasmid, and this transcriptional repression correlates with the Ty1 element. FLAG-H3 expression in cells with pTy1-H3 was induced in SC-His only, but not in SC-Met (methionine dropout) or SC (complete set of amino acids essential for budding yeast) (Fig. S6). This means that the gene expression accompanying marker activation is severely restricted to the kind of amino acid. We next examined whether the protein expression level was proportional to the integration copy number of the pTy1 plasmid. The pTy1-V plasmid can express Venus, a variant yellow fluorescent protein (YFP), with a FLAG epitope tag at the N-terminus under a synthetic promoter (*Psyn*) and synthetic terminator (*Tguo1*) (Fig. 2b: top)^25–27^. In cells with the pTy1-V plasmid grown in SC-Leu (leucine dropout), FLAG-Venus was rarely detected in cells with a single copy of the pTy1-V plasmid (Fig. 2b bottom: lane 1). In cells with 3 copies of the plasmid, a faint band of FLAG-Venus was detected, and the signal intensity of the Flag-Venus band reached a plateau in cells with both 6 and 13 copies of the pTy1-V plasmid, although FLAG-Venus was rarely detected in cells with 13 copies of pTy1-V plasmids grown in YPD (Fig. 2b bottom: lane 2 to 5). Thus, protein expression levels are proportional to the copy number of pTy1 plasmids. Next, we examined whether the gene transcription and protein expression from the plasmid were linked to Ty1 transcription. Cells with pTy1-V plasmids (10 copies) were cultured in YPD and then released into SC-Leu. Real-time quantitative PCR (RT-qPCR) was used to monitor the mRNA levels of Ty1, FLAG-*Venus*, and *LEU2* (*ACT1* as a reference). Ty1, *LEU2* and *Venus* mRNA levels simultaneously increased after cells were released into SC-Leu (Fig. S7A–C). Similar to the fluctuation of *Venus* mRNA, an immunoblot using an α-FLAG antibody detected the FLAG-Venus protein from 2 h after the cells were released into SC-Leu (Fig. 2c: lane 1 to 4). Thus, FLAG-Venus expression correlated with an increase in Ty1 mRNA together with *LEU2* gene induction. Next, cells with the pTy1-V plasmid were cultured in SC-Leu and then released into YPD. The mRNA levels of Ty1, *LEU2* and *Venus* simultaneously decreased after being released into YPD medium (Fig. S7D–F). The FLAG-Venus protein was also abolished during the time course, but its disappearance was delayed compared to FLAG-*Venus* mRNA (Fig. 3d; lanes 1-4 and S7F), which is due to the robustness of the Venus protein^28^. Thus, the repression of the transcription of the *Venus* gene was also linked to the reduction in Ty1 mRNA, together with the inactivation of the *LEU2* gene. Altogether, gene expression in the pTy1 plasmid is connected by Ty1 mRNA transcription.

Changes in Ty1 transcriptional factors might influence gene transcription in cells with the pTy1 plasmid. The SAGA complex, a general transcriptional activator, the Swi/Snf and ISWI chromatin remodeling complexes, and various other transcriptional regulators are involved in Ty1 transcription^20,29–34^ (Fig. 2f). Gcn5 is a general transcriptional activator, and the deletion of *gcn5* reduces the transcriptional activities of both LTRs of the Ty1 elements and *Psyn* promoter^20,26^. The FLAG-Venus protein was rarely detected in the *gcn5*∆ background during the time course (Fig. 2f; lane 4–6). Gcn4 also regulates Ty1 transcription and binds five potential Gcn4-binding motifs (5′-TGAATG-3′) in the vicinity of the LTR region of the Ty1 element (Fig. 2f). However, the Ty1 mRNA level in *gcn4*∆ cells remains at almost the same level as wild-type cells^34^. In contrast to the reduction in the FLAG-Venus protein in *gcn5*∆ cells (Fig. 2f; lane 4–6), the FLAG-Venus protein was constantly expressed in *gcn4*∆ cells during the time course (Fig. 2f; lane 1–3). These results suggest that Ty1 mRNA transcription controls gene expression in the pTy1 plasmid within the Ty1 element.

Jiang reported that transcription of the in-frame Ty1-gene fusion depends on the LTR in the Ty1 element^23^. The FLAG-*HHT1* gene is in-frame with Ty1 transcription in cells with the pTy1-H3 plasmid. If FLAG-*HHT1* mRNA occupied a part of the Ty1 mRNA, FLAG-*HHT1* could be expressed without its own promoter (i.e., the *TDH3* promoter in the pTy1-H3 plasmid). We tested Flag-H3 expression in both cells with the pTy1-H3 plasmid without the *TDH3* promoter region (pTy1-H3∆p) and cells with the pTy1-H3 plasmid following the marker gene induction (Fig. 3a: top). FLAG-H3 was rarely detected in cells with the pTy1-H3∆p plasmid regardless of the integration copy number of the pTy1-H3∆p plasmid, although FLAG-H3 was detected in cells with pTy1-H3 (Fig. 3a: bottom). This suggests that the expression of FLAG-H3 in the pTy1-H3 plasmid needs its own promoter within the Ty1 element. From this result, we hypothesized that marker gene induction could “open” the Ty1 chromatin structure, which is favorable for gene transcription within the pTy1 plasmid. To demonstrate this hypothesis, we performed Micrococcus nuclease (MNase)-chromatin immunoprecipitation (ChIP) to examine the level of trimethylation of histone H3 on K4 (H3-K4me3) in chromatin surrounding the gene on the pTy1-V plasmid^35,36^. Histone H3-K4me3 is a landmark of active gene transcription and is enriched at the 5′ end of the active gene in budding yeast^35,36^. We compared the level of histone H3-K4me3 on the nucleosome both at the start position of Ty1 (Ty1 500) and 5′ end of the Flag-*Venus* gene (Flag-V (N)) on the pTy1-V plasmid (Fig. 3b; upper model) in cells with pTy1-V plasmids (4 copies) cultured in SC-Leu and SC media. The level of histone H3-K4me3 in both Ty1 500 and Flag-V (N) regions was more significantly increased in the SC-Leu culture than in the SC culture, not in *ACT1* region (Fig. 3b). This means that the repressed chromatin within Ty1 became more favorable for transcription following marker gene activation. If the chromatin structure within Ty1 was a detrimental factor for controlling gene transcription in the pTy1 plasmid, two different gene units set in tandem in the pTy1 plasmid could be synchronously expressed following marker gene induction. The pTy1-V-H3 plasmid harbors the following arrayed genes in tandem: the FLAG-*Venus* gene and FLAG-*HHT1* gene with the *Psyn* promoters and *Tguo1* terminators (Fig. 3c: top). Both FLAG-Venus and FLAG-H3 were detected in cells with the pTy1-V-H3 plasmid grown in SC-Leu, although both proteins were rarely detected in YPD (Fig. 3c: bottom). The Flag-Venus level was lower than Flag- H3 (Fig. 3c: bottom) because Flag-H3 can be incorporated into chromatin after the replication fork passes^37^ and the half-life of nucleosomal Flag-H3 extends longer than that of free Flag-Venus (Fig. 2d)^38^. Altogether, marker gene induction can alter the Ty1 chromatin environment, making it more favorable for gene transcription from the pTy1 plasmid.

**Figure 4:**
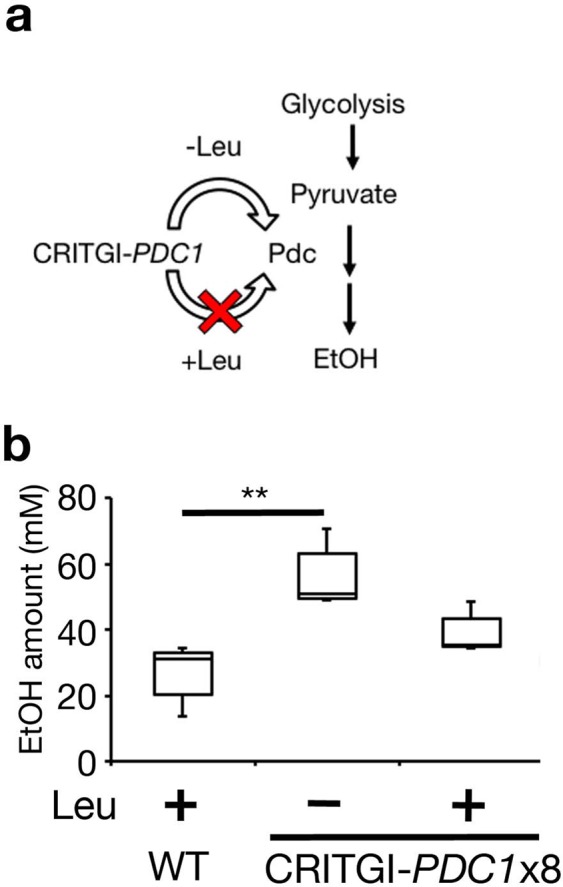
CRITGI can control ethanol production. (**a**) Pdc1 encoding pyruvate decarboxylase is involved in the conversion of pyruvate to ethanol during alcoholic fermentation. The *PDC1* gene is induced in the absence of leucine and repressed in the presence of leucine in cells harboring the pTy1-Pd plasmid containing *PDC1* (CRITGI-*PDC1*). (**b**) Ethanol production was increased in HMY1504 (wild-type cells with the pTy1-Pd plasmid (I. P. No. = 8)) in the presence of Leu. The WT (BY4742) and HMY1504 strains were cultured at 25 °C for 24 h either in SC-Leu (−Leu) or SC + com (+Leu). *P < 0.05. Repeated measures ANOVA with Bonferroni correction. Error bars represent the standard deviation of three biological replicates.

We tested whether CRITGI could be applicable for metabolic engineering. Pyruvate decarboxylase (Pdc) plays an essential role in producing ethanol from pyruvate (Fig. 4a). Additional Pdc expression can increase ethanol production^39^. The pTy1-Pd plasmid possesses the *PDC1* gene, a major gene among the *PDC* gene family, with the *Psyn* promoter and *Tguo1* terminator and the *LEU2* marker gene. Cells with the pTy1-Pd plasmid were cultured in SC-Leu (−L) and SC (+L) for 24 h and with wild-type (BY4742) cells in SC. The amount of ethanol released into the medium by cells with the pTy1-Pd plasmid (−L) increased more than wild-type cells, although the ethanol level in cells with the pTy1-Pd plasmid (+L) was almost similar to that in wild-type cells (Fig. 4b). These data demonstrate that the CRITGI system can produce the metabolite of interest by using amino acids.

In this research, we have developed a novel gene expression system named CRITGI. CRITGI can control gene expression in the pTy1 plasmid integrated into Ty1 by using marker gene activation. For maintaining plasmid in budding yeast, we usually use genes involved in amino acid syntheses and nucleotide synthesis as plasmid markers^18^. Together with conventional molecules that drive inducible promoters, for example, galactose for the *GAL1/1*0 promoter^40^, cupper for the *CUP1* promoter^41^ and tetracycline for the *tet* promoter^42^, various amino acids or nucleotides are available to control gene expression by CRITGI, as long as the genes involved in their syntheses are applicable for the pTy1 plasmid marker genes. CRITGI uses CRISPR/Cas9 to integrate multiple copies of a plasmid into Ty1 loci. The integration number of a plasmid is proportional to the protein expression level. These advantages of CRITGI contribute to metabolic engineering. Fine-tuning the timing and levels of the expression of enzymes involved in both natural and engineered pathways can relieve bottlenecks and minimize the metabolic burden of chemical production^39,43,44^. The production of an end product requires the complete set of metabolic pathways of interest. The incomplete set of the metabolic pathway of interest, which lacks any enzyme steps, might cause the abnormal accumulation of metabolic intermediate(s) harmful for cell survival. CRITGI can induce multiple enzyme-encoding genes that are composed of the metabolic pathway and control the expression levels of enzymes. Although further development of the CRITGI system is needed in the future, CRITGI, which can express a set of enzymes of a metabolic pathway of interest, will contribute to industries such as metabolic engineering, materials engineering, and pharmaceuticals.

Native promoters in *Saccharomyces cerevisiae* regulated by carbon sources^45,46^ or the availability of specific nutrients^47,48^ are applicable to express or repress genes of interest in a trans-acting factor-dependent manner. CRITGI has a unique gene induction system; marker gene activation evokes Ty1 transcription, which is necessary to induce a gene on pTY1plasmid. We favor that Ty1 transcription alters the chromatin structure favorable for transcription of a gene from the pTy1 plasmid. (1) Genes on the pTy1 plasmid need their own promoters. (2) MNase-ChIP shows that H3-K4me3 accumulates at 5′ end of a gene in pTy1 following marker gene activation. (3) Two genes that are set in tandem on the pTy1 plasmid can be synchronously expressed. Swi/Snf and ISWI chromatin remodeling complexes are involved in Ty1 mRNA transcription from the LTR together with transcriptional factors, such as Gcn5^20,33^, suggesting that chromatin remodeling factors may create a chromatin structure favorable for gene transcription from the pTy1-gene plasmid. CRITGI is a unique gene regulation system that alters the Ty1 chromatin structure from repressed to competent allowing gene transcription. Further efforts need to reveal the mechanism to create a chromatin environment favorable for gene transcription within the Ty1 element in the future.

## Methods

### Strains and media

The genotypes of the strains, plasmids and primers used in this study are listed in Table S1. The parental budding yeast strain used in the present study was BY4742 (*MATα his3*Δ *leu2*Δ*1 met15*Δ*0 ura3*Δ*0*)^49^. A yeast strain harboring a single gene deletion was commercially available from the haploid yeast open reading frame deletion collection^50^ (GE Dharmacon, Lafayette, CO, USA). Yeast cells were routinely grown at 30 °C in YPD (1% yeast extract, 2% peptone, 2% glucose) or appropriate synthetic complete (SC) medium^51,52^. If necessary, the media were solidified by including 2% agar. For the time course analysis, yeast cells were cultured at 25 °C. A standard method was used for isolation of the yeast genome DNA^52^. *E*. *coli* strain DH5α^53^, and standard media and methods were used for plasmid manipulations^54^. For the isolation of plasmid free of *dam* methylation, INV110 *E*. *coli* competent cells (Thermo Fisher Scientific, Waltham, MA, USA) were used.

### Plasmid construction

To construct plasmid PHM661 (pTy1), the ~ 870 bp PCR product (Ty1 homologous recombination (HR) sequence, ranging from 1681 bp to 2541 bp in *YPRWTy1-3*) obtained using BY4742 genomic DNA as a template, with forward primer (HMP1196) and reverse primer (HMP1197), was digested with *Bam* HI and *Hind* III and ligated into *Bam* HI/*Hind* III-digested YIplac128 plasmid^55^. For the construction of plasmid PHM688, the *Bam* HI/*Hind* III-digested Ty1 PCR fragment was ligated into the *Bam* HI/*Hind* III-digested pRS403 plasmid^56^.

To construct the plasmid encoding Cas9 and crRNA, we used pML104 plasmid (gift from John Wyrick (Addgene plasmid # 67638)) as a base plasmid^57^. This plasmid requires the insertion of the 20-nt guide sequence within a single guide RNA (sgRNA) cassette. The construction of the sgRNA cassette in the pML104 plasmid was described elsewhere^6,57,58^. The target sequence was set within the Ty1 HR sequence for the guide RNA (gTy1 #3) (5′-ACGTCTTAGAACGGTCTGACGG-3′ (underline: PAM sequence)) and set within the short Ty1 HR sequence for gTy1 #4 (5′-ACCTACATACTGACATATTTGG-3′). To construct the 20-bp guide sequence within the sgRNA, two DNA primers (HMP1192 and HMP1193 for gTy1 #3; HMP1194 and HMP1195 for gTy1 #4) were mixed at a final concentration of 10 μM in annealing buffer (40 mM Tris-Cl pH 8.0, 20 mM MgCl_2_ and 50 mM NaCl). The mixture was incubated at 95 °C for 5 min and cooled down for 90 min until it reached 25 °C. To obtain pML104 plasmid free of *dam* methylation because Dam methylation blocks *Bcl* I digestion, the pML104 plasmid was transformed into a *dam- E*. *coli* competent cell (INV110). To construct the PHM663 (gTy1 #3) and PHM664 (gTy1 #4) plasmids, 20-bp double strand DNA cassettes (gTy1 #3 and gTY1 #4) were ligated into *Bcl* I/*Swa* I-digested pML104 plasmid. The ligation mixture (2.5 µl of 25 ng/µl pML104, 0.5 µl of the annealed DNA strand, 0.1 µl of 10 units/µl *Bcl* I, 0.1 µl of 10 units/μl *Swa* I, 0.1 µl of Quick ligase, 1x T4 DNA ligase buffer (New England Biolabs, Ipswich, MA)) was run for 3 cycles of 5 min at 37 °C, 10 min at 16 °C. Two µl of the ligation mixture was transformed into DH5α *E*. *coli* competent cells to obtain the plasmids (PHM663 and PHM664). Construction of other plasmids is described in Supplementary method.

### Yeast transformation for CRITGI

Yeast transformation was described elsewhere^59,60^. Yeast cells were grown on YPD solid plates, collected with toothpicks and suspended in 100 µl of one-step buffer containing a plasmid mixture^59^. The mixture with 0.5~1.0 µg of integration plasmid (*LEU2* or *HIS3* marker gene) and 0.5~1.0 µg of Cas9/gRNA plasmid (*URA3* marker) was used for transformation. The cell suspension was incubated at 42 °C for 1 h and then plated on SC uracil and either leucine or histidine dropout medium (SC-Ura-Leu or SC-Ura-His). The transformants, plasmids, and parent strains are listed in Table S1. We routinely used the HMY1448 and HMY1459 strains, which have multiple copies of the PHM661 (*LEU2*) and PHM668 (*HIS3*) plasmids already integrated in Ty1 loci, respectively. These strains are useful in obtaining transformants harboring multiple copies of plasmids by transformation.

The isolation of chromosomal DNA from yeast transformants was described elsewhere^52^. To measure the number of plasmids integrated into Ty1 loci, we used the LightCycler 480 SYBR Green I Master (Roche Life Science, Penzberg, Germany). The mix contained 10 µl of 2x LightCycler 480 SYBR Green I Master Mix, 2 µl of 5 µM PCR forward primer, 2 µl of 5 µM PCR reverse primer and ~10 ng of total DNA (final volume 20 µl). Reactions were run for 1 cycle of 10 min at 95 °C; 45 cycles of 20 sec at 95 °C, 20 sec at 53 °C and 20 sec at 72 °C; 1 cycle of 60 sec at 65 °C and 1 sec at 95 °C using either a LightCycler 480 System II or LightCycler Nano (Roche Life Science, Penzberg, Germany). To calculate the plasmid integration number, the *LEU2* gene and *HIS3* gene on the plasmid were compared to the *ACT1* gene as one copy. PCR primers are listed in Table S1.

### Ethanol quantification assay

The cells were cultured overnight in YPD medium at 25 °C. Cells (1 × 10^7^ cells/ml) were resuspended in SC-Leu or SC (+Leu) media and then cultured at 25 °C with shaking for 24 h. An aliquot (1 ml) of the culture was sampled, and the medium and cell pellet were separated by centrifugation. The medium was transferred to a new Eppendorf tube and heated for 5 min at 70 °C to inactivate the enzymes that consume ethanol in the medium. The heat-treated medium (10 µl) was assayed using the Ethanol Colorimetric/Fluorometric Assay Kit (BioVision, Milpitas, CA, USA) according to the manufacturer’s instructions. Fluorescence changes (Ex/Em = 535/587 nm) were monitored using an Infinite F200 fluorescence microplate reader (TECAN, Männedorf, Switzerland). At least three replicates were analyzed for each strain.

### RNA isolation and real time (RT)–PCR

Total RNA was isolated from budding yeast using the RNeasy Mini Kit (Qiagen, Santa Clarita, CA, USA). The relative comparison of the mRNA amount was performed using a One Step SYBR PrimeScript PLUS RT-PCR Kit (Takara-Bio, Kusatsu, Shiga, Japan). The mix contained 10 µl of 2x One Step SYBR RT-PCR Buffer 4, 1.2 µl of Takara Ex Taq HS Mix, 0.4 µl of PrimeScript PLUS RTase Mix, 0.8 µl of 10 µM PCR forward primer, 0.8 µl of 10 μM PCR reverse primer and 100 ng of total RNA (final volume 20 µl). Reactions were run for 1 cycle of 5 min at 42 °C; 1 cycle of 10 sec at 95 °C; 40 cycles of 5 sec at 95 °C, 20 sec at 55 °C; 1 cycle of 1 sec at 95 °C; 1 cycle of 15 sec at 65 °C and 1 sec at 95 °C using either a LightCycler 480 System II or LightCycler Nano (Roche Life Science, Penzberg, Germany). The amount of each mRNA was compared with the amount of *ACT1* mRNA. PCR primers are listed in Table S1.

### Western blotting

Yeast cells expressing FLAG-tagged protein were used. The protein extraction was described elsewhere^61^. Proteins were separated by 15% sodium dodecyl sulfate-polyacrylamide gel electrophoresis (SDS-PAGE) and transferred to nitrocellulose membranes (Amersham Protran) (GE healthcare, Little Chalfont, Buckinghamshire, England). The protein level in each SDS-PAGE lane was normalized and then confirmed using 0.1% Ponceau S solution (Sigma-Aldrich, St. Louis, MO, USA). Mouse monoclonal anti-FLAG M2 antibody (1:1000 dilution) (Sigma-Aldrich, St. Louis, MO, USA) was used to detect FLAG-tagged protein.

### Micrococcus nuclease-chromatin immunoprecipitation (MNase-ChIP) assay

HMY1494 cells (4 copies of PHM820 plasmid) were cultured overnight in 20 ml of YPD medium at 25 °C. Cells (3 ml) were grown either in SC-Leu or SC media shaking at 25 °C for 5 h (OD600: 0.7~0.9). Cells were fixed with formaldehyde at a final concentration of 1% for 15 min. Cell pellets were harvested, washed with TBS buffer (20 mM Tris-Cl pH7.5, 150 mM NaCl), and conserved at −20 °C until use. The cell pellets were subjected to zymolase treatment to make spheroplast, MNase digestion, and chromatin immunoprecipitation^35,36^. Monoclonal antibody to recognize trimethylated histone H3 on lysine 4 (H3-K4 me3) (04-745, Merk Millipore, Burlington, MA, USA) was employed for ChIP^36^. The LightCycler 480 SYBR Green I Master (Roche Life Science, Penzberg, Germany) was used to calculate the percent of input for ChIP for histone H3-K4 me3. The mix contained 10 µl of 2x LightCycler 480 SYBR Green I Master, 2 µl of 5 µM PCR forward primer, 2 µl of 5 µM PCR reverse primer and ~10 ng of total DNA (final volume 20 µl). Reactions were run for 1 cycle of 10 min at 95 °C; 45 cycles of 20 sec at 95 °C, 20 sec at 53 °C, and 20 sec at 72 °C; 1 cycle of 60 sec at 65 °C and 1 sec at 95 °C using LightCycler Nano (Roche Life Science, Penzberg, Germany). The Ty1 500, Flag-*Venus* (N) and *ACT1* regions were used to monitor the status of histone H3-K4 me3. PCR primers are listed in Table S1. The ratio of the percent of input for ChIP in the SC-Leu culture over the SC culture was calculated.

## Supplementary information

Supplementary information

Table S1
