# Supplementary material for: CRISPR/Transposon gene integration (CRITGI) can manage gene expression in a retrotransposon-dependent manner
Source: Sci Rep. 2019 Oct 25;9:15300. doi: 10.1038/s41598-019-51891-6 (PMC6814769; doi:10.1038/s41598-019-51891-6)
Supplement: Supplementary file 1 — Supplementary information [file 41598_2019_51891_MOESM1_ESM.pdf]

Supplementary information

Title: CRISPR/Transposon gene integration (CRITGI) can manage gene expression in a retrotransposon-dependent manner

Authors: Miki Hanasaki[1] and Hiroshi Masumoto[1]*

Affiliations:

[1]Biomedical Research Support Center (BRSC), Nagasaki University School of Medicine, 1-12-4 Sakamoto, Nagasaki, Nagasaki, 852-8523, Japan

*Correspondence to: himasumo@nagasaki-u.ac.jp

Supplementary Methods

DNA manipulation and PCR method

Plasmid DNA was isolated from *E. coli* using a QIAquick Spin Miniprep kit (Qiagen, Santa Clarita, CA, USA). DNA fragments from polymerase chain reaction (PCR) samples or agarose gels were isolated using Wizard SV Gel and PCR Clean-up kit (Promega, Madison, WI, USA). Oligonucleotides were purchased from either Invitrogen (Invitrogen, Carlsbad, CA, USA) or FASMAC (FASMAC, Kanagawa, Japan).

DNA for plasmid construction was generated by PCR using the iProof High-Fidelity DNA polymerase (Bio-Rad Laboratories, Hercules, CA, USA). The mix contained 10 µl of 5x iProof buffer, 0.25 µl each of 100 µM PCR primer, 1 µl of 10 mM dNTP mix, 0.1 µg of template DNA and 0.5 µl of iProof Taq polymerase (final volume 50 µl). Reactions were run for 1 cycle of 10 sec at 98°C; 25 cycles of 10 sec at 98°C, 10 sec at 55°C, and 1 min/kb of desired product at 72°C; 1 cycle of 5 min at 72°C.

Plasmid construction

To construct the PHM740 plasmid, the ~ 370 bp PCR fragment (short Ty1 HR fragment for gRNA #4, ranging from 2177 bp to 2541 bp in *YPRWTy1-3*)) using PHM661 plasmid as a template, with forward primer (HMP1196) and reverse primer (HMP1204), was digested with *Sal* I and *EcoR* I and ligated into *Sal* I/*EcoR* I-digested pRS403 plasmid. To construct the PHM760 plasmid, the ~ 370 bp DNA fragment (short Ty1 HR fragment for gRNA #4) was obtained from PHM661 by *Hind* III/*Sal* I digestion and ligated into the *Hind* III/*Sal* I-digested pRS405 (PHM760) plasmid.

To construct the PHM745 plasmid, the ~ 400 bp PCR product (*TDH3* promoter) obtained using BY4742 genomic DNA as a template, with forward primer (HMP1288) and reverse primer (HMP1329), was digested with *Bam* HI and *Xba* I and ligated into *Bam* HI/*Xba* I-digested PHM740 plasmid. Although the *TDH3* promoter and the Ty1 HR sequence fragment were not compatible in the plasmid, the plasmid harboring the *TDH3* promoter and the short Ty1 HR sequence for gRNA #4 could be constructed. To construct the PHM757 plasmid (pTy1-H3), the ~460 bp PCR product (the FLAG-*HHT1*) obtained using PHM11 as a template, with forward primer (HMP1322) and reverse primer (HMP80), was digested with *Xba* I and *Sac* I and ligated into *Xba* I/*Sac* I-digested PHM745 plasmid. To construct PHM829 plasmid (pTy1-

H3 Δp), PHM757 plasmid was digested with *Bam* HI and *Xba* I to delete *TDH3* promoter fragment, and treated with T4 DNA polymerase to form blunt ends, and self-ligated. The sequence of ligation position was confirmed by direct sequencing.

To construct the synthetic promoter (*Psyn*) [1], the PCR fragment (first part of *Psyn*) obtained using the forward primer (HMP1332) and reverse primer (HMP1333), and the PCR fragment (second part of *Psyn*) obtained using the forward primer (HMP1334) and reverse primer (HMP1335), were digested with *Spe* I and ligated to each other. The PCR fragment (the complete *Psyn*) obtained using the ligation mixture (the first part + the second part of *Psyn*) as a template, with forward primer (HMP1332) and reverse primer (HMP1335), was digested with *Xba* I. To construct the synthetic terminator *Tguo1* [2], the PCR fragment (synthetic terminator *Tguo1*) obtained using the ligation mixture as a template, forward primer (HMP1336) and reverse primer (HMP1337), was digested with *Not* I. The PCR fragment (*E. coli DpaA* gene) obtained using DH5a genomic DNA as a template with forward (HMP1261) and reverse primer (HMP1268) was digested with *Sac* I and *Not* I and ligated with the *Sac* I–digested *Psyn* DNA fragment and *Not* I-digested *Tguo1* DNA fragment. To construct the PHM797 plasmid, the PCR fragment (*Psyn*+*DpaA*+*Tguo1*) obtained from the ligation mixture (*Psyn*+*DpaA*+*Tguo1*) as a

template, with forward primer (HMP1332) and reverse primer (HMP1337), was digested with *Bam* HI and *Sac* I and ligated into the *Bam* HI/*Sac* I-digested PHM760 plasmid.

To construct the PHM820 plasmid (pTy1-V), the ~720 bp PCR product (FLAG-*Venus*) obtained using PHM422 as a template, with forward primer (HMP1322) and reverse primer (HMP1364), was digested with *Xba* I and *Not* I and ligated into *Xba* I/*Not* I-digested PHM797 plasmid to replace the *DpaA* DNA fragment. To construct the PHM833 plasmid (pTy1-Pd), the *Xba* I/*Not* I-digested *PDC1* DNA fragment were ligated into the *Xba* I/*Not* I-digested PHM797 plasmid.

To construct the PHM821 plasmid (pTy1-H3-V), we used the Gold Gate Assembly Protocol with a modification to ligate the FLAG-*Venus* and FLAG-*HHT1* genes in tandem into the plasmid [3-5]. The PCR product (*Psyn*+ FLAG-*Venus*+*Tguo1*) obtained using PHM820 as a template, with forward primer (HMP1369) and reverse primer (HMP1372), and the PCR product (*Psyn*+ FLAG-*HHT1* +*Tguo1*) obtained using PHM815 as a template, with forward primer (HMP1373) and reverse primer (HMP1374), were digested with *Bsa* I. These PCR fragments exposed the designer overhangs as follows: 5’-AAAA- *Psyn*+ FLAG-*HHT1* +*Tguo1*-CTCA-3’ and 5’-CTCA- *Psyn*+ FLAG-*Venus* +*Tguo1*-ACTG-3’ (note that all overhangs are

listed here as top-strand sequences for clarity). These two PCR fragments were ligated into *Bsa* I-digested PHM802 plasmid (5'-ACTG-vector-AAAA-3'). The ligation mixture (40 fmol of each DNA fragment and 1x Takara Ligation kit ver. II (Takara-Bio, Kusatsu, Shiga, Japan)) was incubated for 30 min at 16°C. Two μl of the ligation mixture was transformed into DH5α *E. coli* competent cells to obtain the plasmid (PHM821).

Pulse-field gel electrophoresis (PFGE) analysis

The equivalent amounts of BY4742 and HMY1448 strains (OD_{600}=0.8) used for PFGE analysis. The preparation of agarose plug containing yeast chromosomal DNA was described elsewhere [6]. The PFGE analysis (CHEF-DR II system (Bio-Rad Laboratories, Hercules, CA, USA) using prepared DNA sample was performed following to the manufacturer's instruction.

Supplementary references

1 Redden, H. & Alper, H. S. The development and characterization of synthetic minimal yeast promoters. *Nat Commun* **6**, 7810, doi:10.1038/ncomms8810 (2015).

2 Curran, K. A. *et al.* Short Synthetic Terminators for Improved Heterologous Gene Expression in Yeast. *ACS Synth Biol* **4**, 824-832, doi:10.1021/sb5003357 (2015).

3 Agmon, N. *et al.* Yeast Golden Gate (yGG) for the Efficient Assembly of S. cerevisiae Transcription Units. *ACS Synth Biol* **4**, 853-859, doi:10.1021/sb500372z (2015).

4 Lee, M. E., DeLoache, W. C., Cervantes, B. & Dueber, J. E. A Highly Characterized Yeast Toolkit for Modular, Multipart Assembly. *ACS Synth Biol* **4**, 975-986, doi:10.1021/sb500366v (2015).

5 Guo, Y. *et al.* YeastFab: the design and construction of standard biological parts for metabolic engineering in Saccharomyces cerevisiae. *Nucleic Acids Res* **43**, e88, doi:10.1093/nar/gkv464 (2015).

6 Maringele, L. & Lydall, D. Pulsed-field gel electrophoresis of budding yeast chromosomes. *Methods Mol Biol* **313**, 65-73, doi:10.1385/1-59259-958-3:065 (2006).

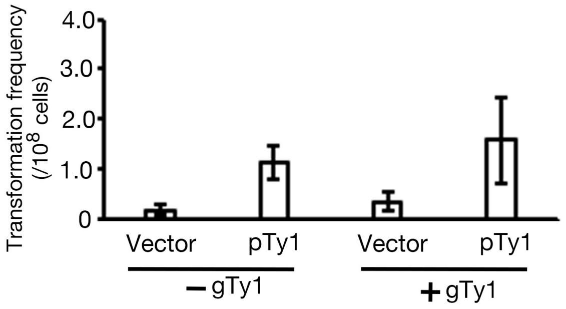

Fig. S1. HR Ty1 sequence is required for transformation in CRITGI. YIplac128 was used as "vector".

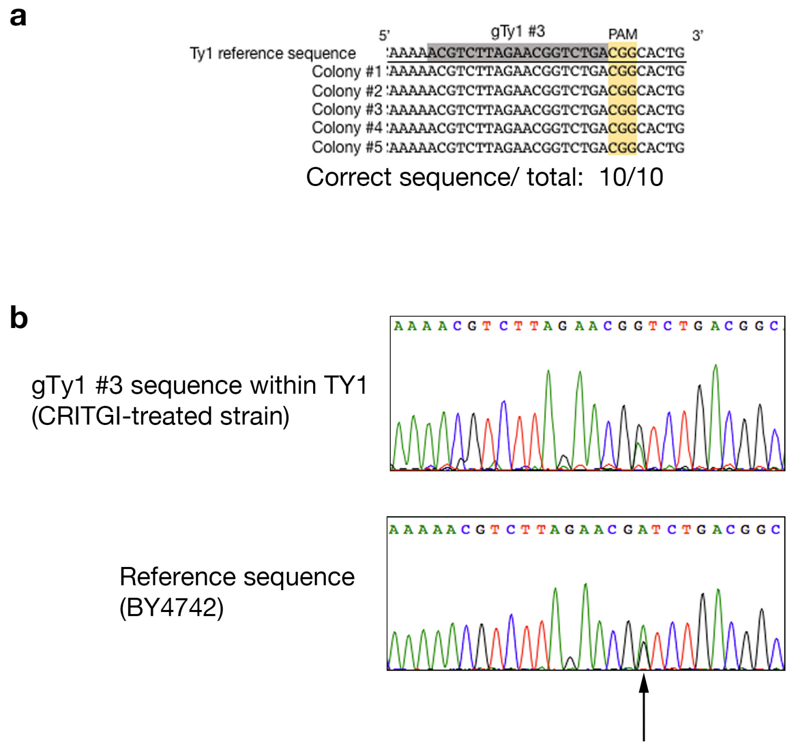

Fig. S2. pTy1 plasmid correctly integrates into Ty1 locus. (a) the representative data analyzing the sequence around gTy1 #4 (5 samples). The sequences among all samples (n=10) were identical to the reference sequence of parent cell (BY4742). Purple sequence: PAM sequence. (b) The sequence patterns around gTy1 #4 between transformant and parent cell (BY4742). The arrow indicates the A and T are mixed at this position of Ty1 which has not been reported in BY4742 strain in the database (*Saccharomyces* genome database).

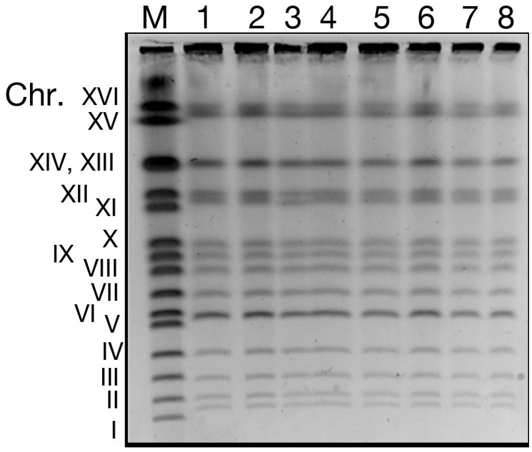

Lane M: Budding yeast chrosome marker (Biorad)
1: BY4742 chromosome
2-8: CRITGI-transformants

Fig. S3. CRITGI cause neither the translocation or loss of the chromosome arm.

HMY1448 strains (various number of PHM661 plasmids in Ty1 loci) were used. Whole Each chromosome of budding yeast (I~XVI) were separated by pulse-field gel electrophoresis (PFGE). Chr.: chromosome. M: DNA Size Markers-Yeast Chromosomal (Bio-Rad Laboratories, Hercules, CA, USA)

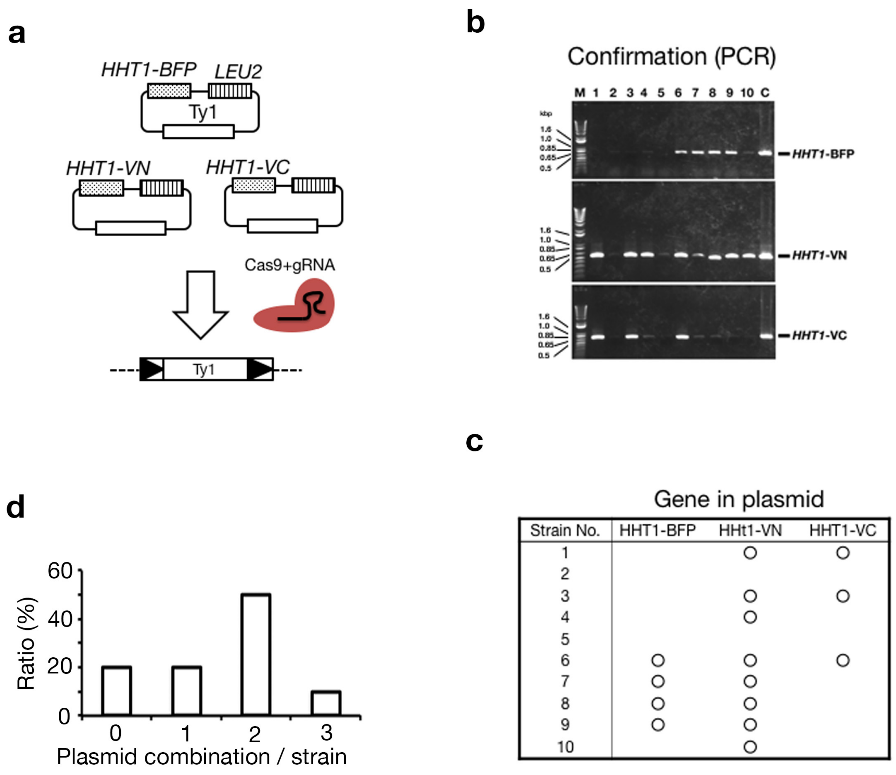

Fig. S4. Multiple combination of different pTy1 plasmids can be integrated by CRITGI.

(a) Three types of pTy1 plasmids (PHM765, PHM766 and PHM767) with PHM664 were simultaneously transformed into HMY1448 strain. (b) Each plasmid integration was confirmed by PCR. PCR products were analyzed in 1% agarose gel electrophoresis, and detected by ethidium bromide. M: 1 kbp plus DNA ladder (Thermo Fischer Scientific, Waltham, MA, USA). C: positive control band showing each epitope-tagging *HHT1* gene. (c) Diagram to show the gene in integrated in transformants. *HHT1*-BFP in PHM765 plasmid, HHT1-VC in PHM766

and HHT1-VN in PHM767. (d) The graph indicates the percentage of plasmid combination in transformants.

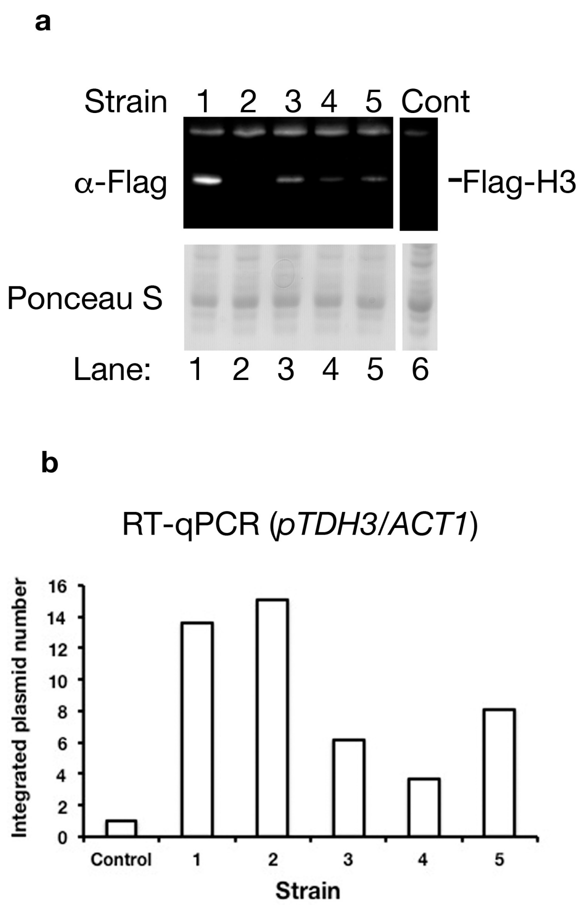

Fig. S5. The Flag-histone H3 expression in cells with the pTy1-H3 plasmid. (a) HMY1466 cells (wild type cell with pTy1-H3 plasmid) (n=5) were cultured in YPD at 25˚C overnight. Cell extracts were analyzed by immunoblot using α-Flag antibody. Control: BY4742 strain expressing no Flag-tagging histone H3. (b) Integrated plasmid numbers (I. P. No.) in HMY1466 cells were calculated by the ratio of *TDH3* promoter (in pTy-H3 plasmid) for *ACT1* gene as 1 copy using RT-PCR.

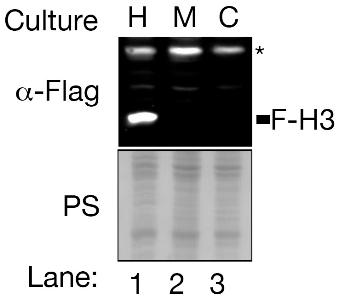

Fig. S6. FLAG-H3 expression requires activation of the marker gene in the pTY1-H3 plasmid, but not simple medium exchange to SC medium. HMY1466 strain (wild type cell with pTy1-H3 plasmid (I. P. No. =11)) was cultured at 25˚C overnight in each culture medium. Cell extracts were analyzed by immunoblot using α-Flag antibody. M: SC-Met. C: SC.

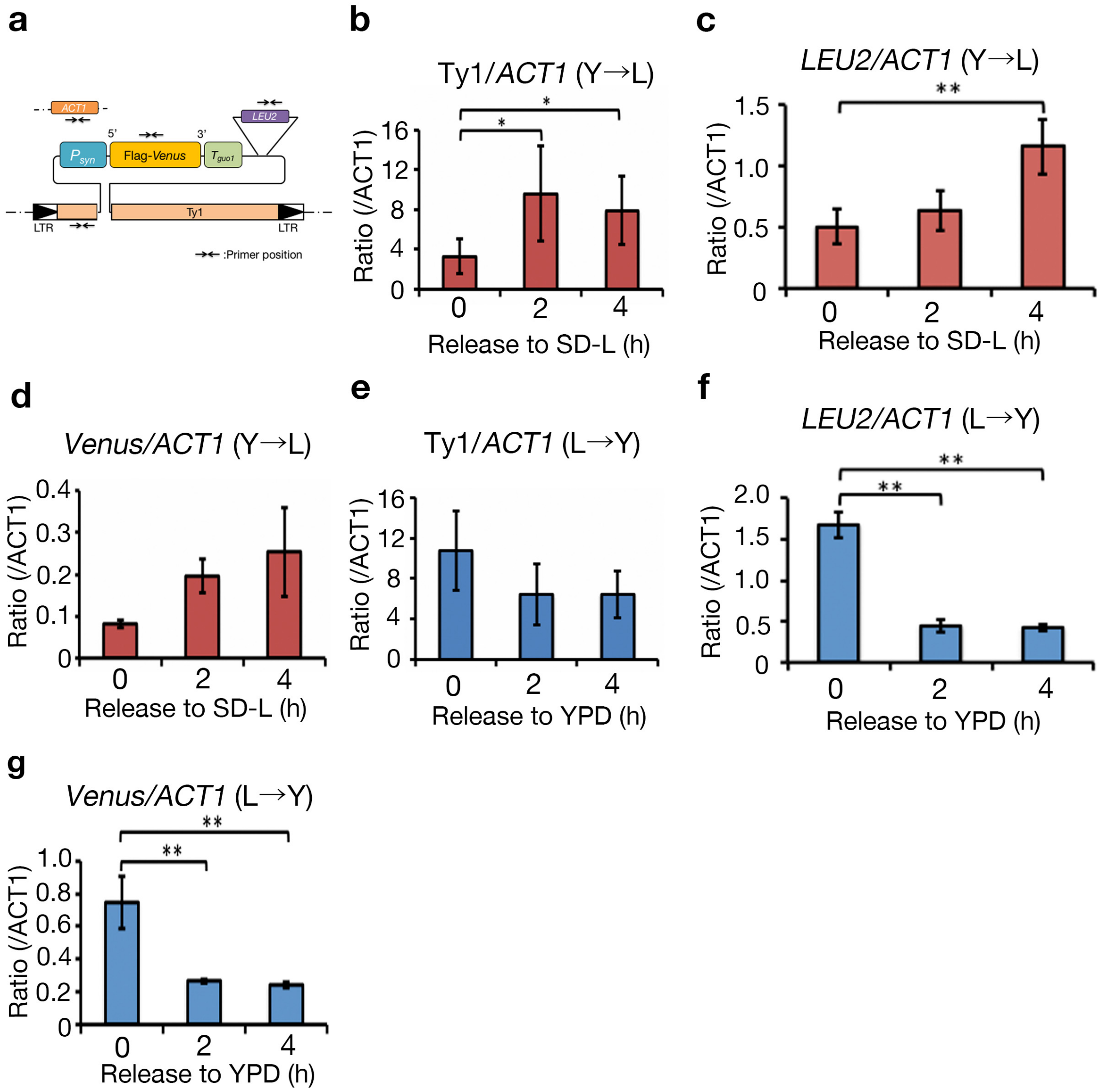

Fig. S7. RT-qPCR analysis to measure the mRNA levels of Ty1, *LEU2* and *Venus* genes.

mRNA was relatively measured with *ACT1* mRNA. (a) the position of primers for RT-qPCR analysis (▶◀). (B to D) HMY1476 strain (wild type cell with pTy1-V plasmid (I. P. No. =10)) was cultured in YPD, and then released into SC-LEU (Y→L). Aliquots were picked up according to the time course. Y: YPD. L: SC-Leu. (b) Ty1 mRNA, (c) *LEU2* mRNA and (d)

Venus mRNA. (E to G) HMY1476 cells were cultured in SC-Leu, and then released into YPD (L→Y). Aliquots were picked up according to the time course. (e) Ty1 mRNA, (f) *LEU2* mRNA and (g) *Venus* mRNA. **P*<0.05. ***P*<0.01. Repeated measures ANOVA with Bonferroni correction. Error bars represent standard deviation of three biological replicates.

Table S1. Yeast strains, plasmids, primers, sequences of the promoter, the terminator, Ty1 HR sequence and Short Ty1 HR sequence used in this study.
